# Modeling unobserved heterogeneity in multistate event history data using frailty and weighted survival approaches

**DOI:** 10.1038/s41598-025-30535-y

**Published:** 2025-12-10

**Authors:** Abhipsa Tripathy, Gajendra K. Vishwakarma, Atanu Bhattacharjee

**Affiliations:** 1 Department of Mathematics and Computing, IIT(ISM) Dhanbad, 826004 Dhanbad, Jharkhand India; 2 Population Health and Genomics, School of Medicine, University of Dundee, Dundee, UK

**Keywords:** Multistate model, Censoring, Frailty effect, Heterogeneity, Proportional hazard model, Cancer, Mathematics and computing

## Abstract

Conventional survival analysis models typically assume that the hazard function depends solely on the baseline hazard and covariate values, overlooking unobserved factors that influence survival outcomes. In practice, however, unmeasured variables often contribute to heterogeneity among seemingly similar individuals. Frailty models offer an effective approach to account for such unobserved heterogeneity, providing a robust framework for analyzing naturally clustered survival data. This study applies frailty models to multistate event history data, emphasizing their ability to handle unobserved heterogeneity. We introduce individual-specific survival weights to adjust survival times, better reflecting the impact of unmeasured factors. These weighted survival times are critical when data exhibit bias or when standard models fail to fully capture the influence of investigated variables. Through a simulation study, we evaluate the effectiveness and performance of frailty models in a multistate framework, comparing mean, mean squared error (MSE), and bias of regression coefficients with and without frailty. For example, in the simulated dataset for age bias has reduced from -0.01 in unweighted survival time to -0.03 in weighted survival time for transition $\tau _{12}$, similarly for $\tau _{23}$ bias has reduced from 0.01 to -0.05. Our findings underscore the importance of addressing unobserved heterogeneity in survival analysis, particularly in multistate models with weighted survival times.

## Introduction

Frailty models and multistate models (MSMs) are powerful tools used in survival analysis to handle heterogeneity among individuals and to better represent complex processes involving multiple intermediate states between health and death. Frailty refers to unobserved, random effects that account for differences in survival rates among individuals that cannot be explained solely by measured covariates, while MSMs extend survival models to allow transitions between multiple states over time. This joint application help in understanding not just whether an event will happen, but when and how individuals move between various health states before reaching a terminal state.

Frailty models were initially proposed by Vaupel et al.^1^ to explain unobserved heterogeneity in survival times across ostensibly similar individuals. These models frequently extend the Cox proportional hazards (Cox PH) model, which is among the most prevalent models utilised for comparing treatments in survival analysis. However, while the Cox PH model posits that the hazard function can be fully characterized by the baseline hazard and covariate values, it does not account for the unobserved variability or random effects. This is where frailty models are particularly useful. Frailty introduces random effects that adjust for unobserved heterogeneity between individuals or clusters^2^. The Markov assumption in multistate models, which posits that future states depend only on the current state and not on prior transitions, often fails when unobserved effects are not accounted for.

In multistate models, individuals transition through various states (e.g., from healthy to diseased to dead), making them ideal for analyzing chronic diseases that involve multiple stages. Incorporating frailty into MSMs addresses the association between the transition intensities of individuals, as the likelihood of transitioning from one state to another is not independent but often influenced by underlying, unmeasured factors. For example, frail individuals may progress more rapidly through disease stages compared to healthier individuals, and this progression can be modelled with frailty to reflect unobserved individual or group-level variability^3,4^.

Frailty models can be univariate, where each individual is treated independently, or multivariate shared frailty models, where individuals are grouped into clusters and their survival times are correlated. The latter is particularly relevant in MSMs, as individuals often belong to clusters (e.g., patients receiving the same treatment) and exhibit shared risks. The shared frailty model captures this correlation, offering a more nuanced understanding of clustered survival data^5^. Moreover, shared frailty models allow for the inclusion of both fixed effects, which account for observable factors, and random effects, which capture the unobserved variability in survival times.

Applications of frailty models in multistate event history data are well-established, with numerous studies demonstrating their effectiveness in capturing the complex dynamics of survival processes^2,6^. Aalen (1988) proposed a time-homogeneous Markov model with frailty, where the frailty term affects all transitions within the Markov process^7^. Other works have extended this approach to explore random effects across transitions in various medical conditions, such as Bhattacharya and Klein’s work on event state transitions and Yen et al.’s study on adenoma-carcinoma progression in the small bowel^8,9^.

The combination of frailty models with MSMs allows for the analysis of both individual-level and group-level variability. Individual-level variability is reflected in the transitions between states, where frail individuals may advance more quickly through disease stages, while group-level variability captures the differences in survival linked to clusters of individuals (e.g., patients undergoing the same treatment or belonging to the same demographic group). For instance, frailty models help represent how survival times can vary even among patients with similar observable characteristics but differing in their unobserved frailty levels^10–12^.

In this article, we propose a modified survival time that incorporates individual-level survival weights to assess the influence of unobserved variations. Survival times that disregard heterogeneity or frailty may result in biased results due to their failure to consider this unobserved heterogeneity. In survey studies including groups or clusters (e.g., families, ethnic groups), frailty models adjust survival time to account for shared frailty within those clusters. By incorporating frailty into survival time, the model recognizes that individuals with greater frailty encounter events earlier, which is essential for tailored survival analysis. To elucidate this scenario in practical data analysis for the simulation study, we generated a multistate dataset with three parametric baseline hazards exponential, Weibull, and Gompertz and analyzed frailty models for different transitions using a gamma distribution. By applying these models to real data, such as the European Society for Blood and Marrow Transplantation (EBMT) dataset, we demonstrate how frailty models enhance the precision of survival predictions by accounting for random effects and clustering within multistate data. This approach offers deeper insights into the dependencies and assumptions underlying survival processes and how unobserved heterogeneity can impact survival outcomes.

**Fig. 1:**
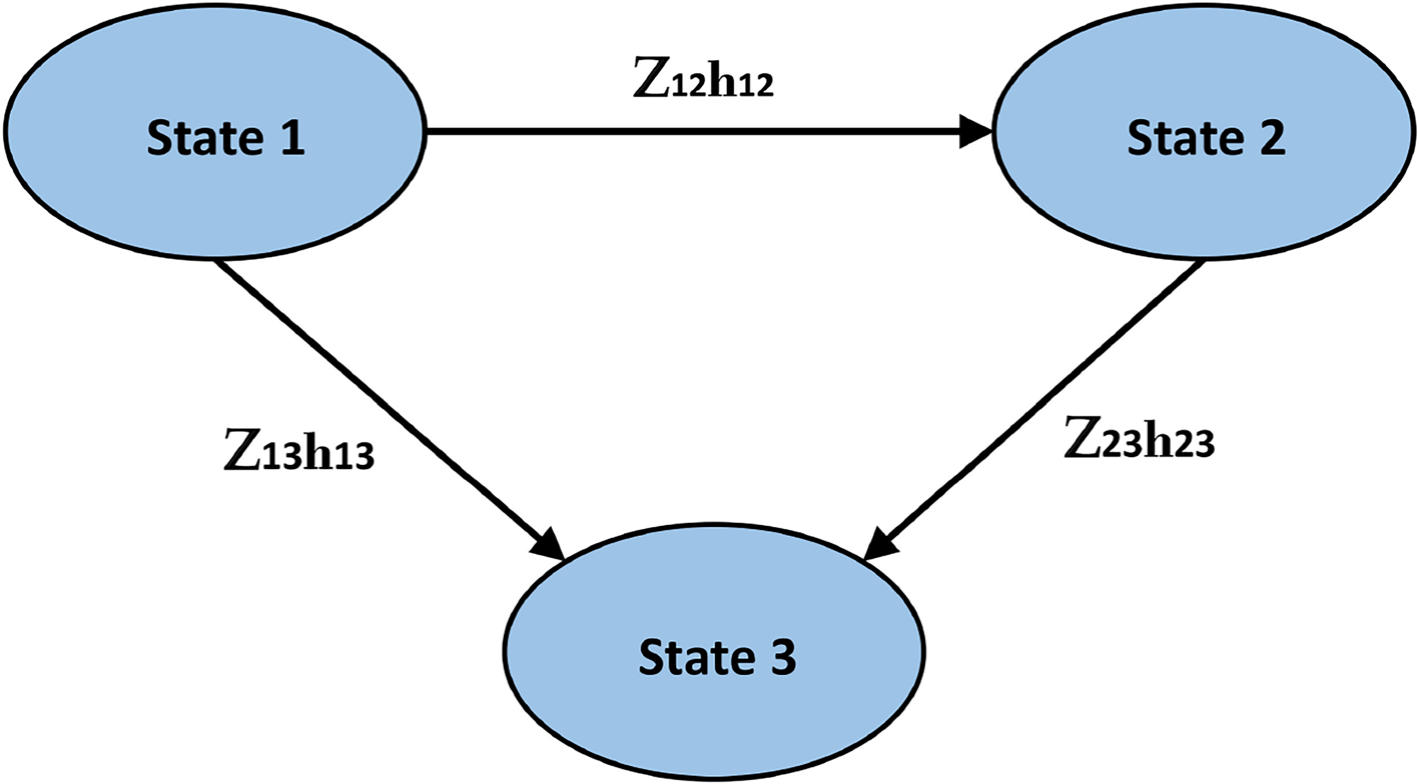
Multi state model with three states, transition-specific frailty terms $Z_{12}, Z_{23}, Z_{13}$, and frailty estimates.

## Methods

### Frailty in survival models

Frailty models play a crucial role in survival analysis by addressing unobserved heterogeneity that affects individuals’ risk of events such as death or disease progression. This unobserved variability, termed *frailty*, accounts for factors such as genetic predispositions or lifestyle influences that are not captured by observed covariates but nonetheless influence survival outcomes. In heterogeneous populations, accounting for frailty enables a more comprehensive understanding of survival data.

Frailty models are particularly important in the context of *multistate models (MSMs)*, where individuals may transition between multiple health states over time. For instance, in cancer progression studies, patients may transition through stages such as diagnosis, remission, relapse, and death, with timing and likelihood influenced by unobserved factors like genetic susceptibility or treatment response. The timing and likelihood of these transitions can be influenced by unobserved factors such as genetic susceptibility, lifestyle, or individual response to treatment. Incorporating frailty into the MSM framework allows for the modeling of this unobserved heterogeneity, leading to more accurate estimates of transition intensities and improved understanding of the underlying disease dynamics. MSMs extend traditional survival models by tracking not just the occurrence of a single event, but a series of transitions between states (e.g., from “healthy” to “diseased” to “dead”). In real-world scenarios, the hazard of transitioning from one state to another may not only depend on observed covariates but also on unobserved frailty, which introduces individual variability into these transitions.

Let *T* represent the survival time, with the survival function expressed as $S(t) = P(T > t) = 1 - F(t)$, where *F*(*t*) is the cumulative distribution function of *T*. In MSMs, the Cox proportional hazards (Cox PH) model is frequently used to explore the association between covariates and time-to-event through the hazard function, which is described as:

1 $$\begin{aligned} h(t|X)=h_{0}(t)\exp (\beta X) \end{aligned}$$

Here, $h_{0}(t)$ represents the baseline hazard when all covariates *X* are held constant, and $\beta$ represents the coefficients that describe the influence of the covariates on the hazard function. However, in the context of MSMs, populations are often heterogeneous individuals may differ due to health conditions, genetic factors, or lifestyle, all of which may introduce unobserved variability, or frailty.

In multistate processes, individuals transition between states at different rates depending on their frailty. Vaupel et al. (1979) pioneered the concept of frailty in survival models by suggesting that the hazard function is influenced by an unobserved frailty component, such that the hazard for an individual is a product of their frailty *Z* and the baseline hazard rate:

2 $$\begin{aligned} h(t|Z) = Z h(t) \end{aligned}$$

In the above model, *Z* represents the unobserved frailty factor that varies between individuals, influencing their hazard rate. Typically, *Z* is modelled as a latent random variable characterised by a non-negative distribution with a mean of 1, meaning that $h(t|Z=1)$ pertains to the hazard for an individual exhibiting average frailty. In MSMs, frailty introduces individual-specific random effects that impact transition probabilities between states, making the analysis more robust by accounting for unobserved heterogeneity. The proportional hazards model accounted for frailty in the presence of covariates, can be adjusted as:

3 $$\begin{aligned} h(t|Z,\textbf{X})&= h_{0}(t)\exp (\beta X + \omega Z) \nonumber \\&= Z h(t|\textbf{X}=\textbf{x}) \end{aligned}$$

Here, *X* denotes the vector of observed covariates, while $\omega$ represents the unobserved random effects due to frailty. This extended formulation allows the model to incorporate both observed covariate effects and unobserved heterogeneity, improving its accuracy in predicting survival outcomes, especially in multistate settings.

The conditional survival function for an individual with frailty *Z* is expressed as:

4 $$\begin{aligned} S(t|Z) = \exp (-Z H(t)) \end{aligned}$$

Where *H*(*t*) denotes the cumulative hazard function. This equation can be augmented to incorporate both covariates and frailty, resulting in a survival function *S*(*t*|*Z*, *X*) , which indicates the ratio of individuals surviving till time *t*, given both the covariates *X* and frailty *Z*.

#### Marginal hazard and Laplace transformation

In frailty models, the marginal hazard represents the mean hazard across all people, considering the distribution of frailty *Z*. The marginal hazard is calculated as:

5 $$\begin{aligned} h'(t) = E[Z|T\ge t]h(t) \end{aligned}$$

where $E[Z|T\ge t]$ represents the conditional expectation of frailty given survival up to time *t*. The equation 4 indicates that individuals exhibiting greater frailty are more prone to encounter events sooner, resulting in a temporal shift in the distribution of frailty within the population. We can use the Laplace transformation to derive the marginal hazard, a powerful mathematical tool that simplifies handling the frailty distribution.

The Laplace transformation of a non-negative random variable $Z$ is defined as:

6 $$\begin{aligned} \textbf{L}_{z}(c)=E(\exp (-Zc)) \end{aligned}$$

This transformation is useful in survival analysis because it helps link the conditional and marginal hazard functions by capturing the effects of frailty on survival. Now we will define the Laplace transformation of the conditional frailty $Z|T \ge t$ defined as:

7 $$\begin{aligned} L_{Z|T \ge t} (c)&= E[\exp (-Zc)|T \ge t] \nonumber \\&= \frac{L(c+H(t))}{L(H(t))} \end{aligned}$$

This transformation is instrumental in defining the marginal hazard function which is expressed as:

8 $$\begin{aligned} h'(t)=h(t)\left[ \frac{-L^{'}(H(t))}{L(H(t))}\right] \end{aligned}$$

This formulation links the conditional and marginal hazards, making the model more flexible and providing a clearer understanding of how unobserved heterogeneity impacts survival. The mean and variance of frailty for individuals surviving beyond time $t$ can also be derived from the Laplace transformation, offering insights into the expected frailty distribution in the population over time.

By incorporating frailty into multistate models, we account for unobserved heterogeneity that may otherwise skew survival predictions. Frailty models extend the conventional Cox PH model by adding random effects that adjust the hazard function based on unobserved factors. The use of Laplace transformations further simplifies the derivation of marginal hazards, enhancing the model’s flexibility. This integration is particularly critical for studying long-term survival patterns in heterogeneous populations, where unobserved variability plays a significant role in influencing outcomes.

#### Frailty effects in multi state models

Frailty effects capture the unobserved differences between individuals that influence their risk of transitioning between different health states in survival analysis. When MSMs, it is important to note that frailty can significantly affect the transition rates between states, leading to more accurate predictions of events such as disease progression or recovery. MSMs are used to model processes where patients transition between multiple states (e.g., healthy, ill, dead) over time. These models often follow a Markov process, which assumes that future transitions rely exclusively on the present state, rather than the trajectory taken to attain that state^13^.

Let $i$ and $j$ represent two distinct states in an MSM. The probability of moving from state $i$ to state $j$ over time is denoted as:

9 $$\begin{aligned} P_{ij}(s,t) = P(X_t = j \mid X_s = i) \end{aligned}$$

where $P_{ij}(s,t)$ denotes the probability of occupying state $j$ at time $t$, conditional on the individual existing in state $i$ at time $s$. The instantaneous transition rate from state $i$ to state $j$, or the hazard of making that transition, is presented as:

10 $$\begin{aligned} h_{ij}(t|\textbf{X} = \textbf{x}) = \lim _{\Delta t \rightarrow 0} \frac{P_{ij}(t, t + \Delta t)}{\Delta t} \end{aligned}$$

This rate represents how likely an individual is to transition from state $i$ to state $j$ at a specified time.

This study, employs an *illness-death model*, a type of MSM that allows individuals to transition between different health states, typically including a “healthy” state, an “ill” state, and death. For example, in cancer research, a patient might transition from being healthy to developing cancer, and later to death. This model, as depicted in Fig. 1, represents disease progression through three states: 1 (healthy), 2 (ill), and 3 (death). The hazard of transitioning between these states is modeled using a transition-specific Cox proportional hazards (Cox PH) model. This means that for each possible transition (e.g., healthy to ill, ill to death), we estimate a separate hazard function. When incorporating frailty, the hazard of transitioning from state $i$ to state $j$ (where $i \ne j$) is given by:

11 $$\begin{aligned} h_{ij}(t|\textbf{X} = \textbf{x}, Z_{ij}) = h_{0ij}(t) \exp (\beta ^T \mathbf {x_{ij}} + \omega Z_{ij}) = Z_{ij} h_{0ij}(t) \exp (\beta ^T \textbf{x}) \end{aligned}$$

where $h_{0ij}(t)$ is the baseline hazard, which represents the risk of transitioning between states $i$ and $j$ when all covariates $\textbf{x}$ are held constant and no frailty is present. $\mathbf {x_{ij}}$ is the vector of covariates (e.g., age, treatment, genetic factors). $Z_{ij}$ is the frailty term, which accounts for unobserved factors that affect transition rates and $\beta$ and $\omega$ are coefficients representing the effects of observed covariates and frailty, respectively.

For example, in a study of cancer progression, $\textbf{x}$ might include the patient’s age and treatment type, while $Z_{ij}$ could capture unmeasured factors like genetic susceptibility or lifestyle differences that increase the risk of progression to the ill or death state. Figure 1 illustrates a three-state illness-death model with frailty. Transition-specific frailty terms $Z_{12}, Z_{23}, Z_{13}$ represent the unobserved factors influencing the transition rates between the three states: 1 (healthy), 2 (ill), and 3 (death). This structure allows for individual variability, where frailty terms capture the impact of unobserved risk factors on these transitions.

To better understand the role of frailty, we compute the mean and variance of frailty for individuals who have survived beyond time $t$, given their covariate information. These measures describe the distribution of unobserved risk factors affecting the transition rates. For the mean frailty, we use the following formula:

12 $$\begin{aligned} E(Z_{ij} \mid T> t, \textbf{X} = \textbf{x})&= \int z_{ij} f(z_{ij} \mid T > t, \textbf{X} = \textbf{x}) dz_{ij} \nonumber \\&= - \frac{L'(H_{ij}(t))}{L(H_{ij}(t))}. \end{aligned}$$

The variance of frailty is calculated as:

13 $$\begin{aligned} V(Z_{ij} \mid T> t, \textbf{X} = \textbf{x})&= \int (z_{ij} - \mu _{z_{ij}})^2 f(z_{ij} \mid T > t, \textbf{X} = \textbf{x}) dz_{ij} \nonumber \\&= \frac{L''(H_{ij}(t))}{L(H_{ij}(t))} - \left[ \frac{L'(H_{ij}(t))}{L(H_{ij}(t))} \right] ^2 . \end{aligned}$$

#### Markov and semi-Markov processes in multi state models

In MSMs, the *Markov process* assumes that the duration in each state (also called sojourn time) follows an exponential distribution, meaning that the risk of shifting from one state to another is perpetual over time. However, this assumption is frequently impractical in real-world contexts. For example, the probability that a cancer patient transitions from illness to death may increase the longer they remain in the ill state.

To account for this, we use *semi-Markov processes (SMPs)*, which allow for non-exponential waiting times between transitions. Unlike the Markov process, where transition rates are contingent solely upon the present state, SMPs take into account the duration of time spent in the present state before transitioning. This is particularly useful in biomedical studies, where the duration of illness or treatment affects the probability of recovery or relapse^14^. For example, in chronic diseases, the likelihood of transitioning from the ill state to death often depends on how long the patient has been ill. In SMPs, the transition rate between states is contingent upon the time duration already expended in the present state, but not on the path taken to reach it^15^. This makes SMPs more flexible and suitable for modelling processes such as disease progression, recovery, or relapse, where the time spent in intermediate states influences future outcomes^13^.

#### Practical implications of frailty in multi state models

By incorporating frailty and semi-Markov processes into MSMs, researchers can more accurately model complex disease progression and better predict patient outcomes. This approach is particularly valuable in clinical decision-making, where understanding the timing and likelihood of disease transitions can inform treatment strategies and interventions. As discussed by Asanjarani et al. (2021)^13^, incorporating frailty terms into MSMs improves the accuracy of survival predictions by accounting for unobserved heterogeneity.

### Weights on survival time in multi state models

In many real-world applications, such as modeling disease progression, it is essential to account for the time individuals spend in each state before transitioning to the next. While traditional Markov models assume that state transitions are memoryless (i.e., the time spent in a state doesn’t affect the next transition), SMPs provide more flexibility. In an SMP, the duration spent in a state directly influences the likelihood of transitioning to another state. This makes SMPs particularly useful for applications where the timing of events plays a critical role, such as in disease progression models.

To define an SMP in a multistate model, consider a homogeneous Markov chain $J_{n}, n \ge 0$ with finite state space $S = \{1,2,3\}$. The state $J_n$ denotes the state of the system following $n$ transitions. The transition probability for the $n^{th}$ jump from state $i$ to state $j$ is given by:

14 $$\begin{aligned} P_{ij} = P(J_{n} = j | J_{n-1} = i) \end{aligned}$$

where $i,j = 0,1,2,3$ and $i \ne j$. If $T_{0}, T_{1}, T_{2}, \dots$ represent the times at which a particular state is entered, then $0 = T_{0}< T_{1}< T_{2} < \cdots \tau _{n}$ defines an increasing sequence of jump times (i.e., the times at which transitions occur). The stochastic process $J_{n}$ forms an SMP, indicating that when an individual is in state $i$, the subsequent state is $j$ is attained with probability $P_{ij}$ , and the time until the subsequent transition is governed by a stochastic variable characterized by the cumulative distribution function $F_{ij}$ , defined as:

15 $$\begin{aligned} F_{ij}(t) = P(\tau _{n} \le t | J_{n-1} = i, J_{n} = j), \quad t \ge 0, \end{aligned}$$

where $\tau _{n} = T_{n} - T_{n-1}$ is the time spent in the current state before transitioning.

#### Transition probabilities and survival functions

In survival analysis, it is important to not only predict transitions between states but also to understand how the likelihood of these transitions changes over time. A key concept for this is the hazard function, which measures the instantaneous risk of transitioning between states at a specific time.

The hazard function is defined as:

16 $$\begin{aligned} h_{ij}(t) = \lim _{\Delta t \rightarrow 0} \frac{P(\tau _{n} \in (t, t + \Delta t) | J_{n-1} = i, J_{n} = j, \tau _{n} > t)}{\Delta t} \end{aligned}$$

In practical terms, the hazard function $h_{ij}(t)$ represents the instantaneous risk of transitioning from state $i$ to state $j$ at time $t$, given that no transition has occurred prior to $t$. This function is fundamental in survival analysis because it quantifies the likelihood of transitioning between states over time, helping to identify periods of higher or lower transition risk.

The survival function, conversely, provides the probability of remaining in state $i$ for a minimum of $t$ time units. It is defined as:

17 $$\begin{aligned} S_{i}(t) = P(\tau _{n} > t | J_{n} = i) = \sum _{i \ne j} P_{ij} S_{ij}(t) \end{aligned}$$

This function is critical for understanding how long individuals remain in different states before transitioning. By modeling these durations, researchers can gain insights into the dynamics of system states over time.

#### Real-world relevance and examples

In real-world applications like oncological studies, patients may undergo various treatments or experience disease progression at different rates. For example, in a model with three states such as “transplant/surgery” (state 1), “progression” (state 2), and “death” (state 3) understanding how long patients stay in each state before transitioning can provide important insights into disease dynamics.

Using a weighted survival model in this context allows researchers to capture the relative importance of different transitions. For instance, transitions from “progression” to “death” may be more critical to model than transitions from “transplant” to “progression.” This approach helps make more accurate predictions, improve understanding of disease progression, and tailor interventions to patient subgroups. By introducing weights, survival estimates are refined across the population, ensuring that transitions with more clinical relevance receive greater focus.

#### Discussion of weights

In real-world applications, not all transitions are equally important. For example, in oncological studies, a transition from “progression” (state 2) to “death” (state 3) may be more critical to model than a transition from “transplant/surgery” (state 1) to “progression” (state 2). This is where the introduction of weights on survival times becomes advantageous. Weighting survival times allows us to prioritize certain transitions based on their clinical or practical relevance. As demonstrated by Yin et al. (2015)^16^, incorporating weighted survival times can significantly improve predictive accuracy in MSMs, particularly when certain transitions are prioritized over others.

Let $k = 1, 2, \dots , n$ represent the number of individuals undergoing transition $\{ij\}$. The weighted survival time for an individual is defined as:

18 $$\begin{aligned} \hat{S_{k}}(t) = \sum _{i \ne j} w_{ij}^{'} S_{ij}(t) \end{aligned}$$

where $w_{ij}^{'}$ is the weight assigned to a specific transition, and $S_{ij}(t)$ is the corresponding survival time.

#### Transition probabilities in practice

In many clinical scenarios, patients are more prone to transition from an initial state of health (state 1) to a state of illness (state 2) before progressing to death (state 3). For instance, in a chronic disease like cancer, the probability of a patient transitioning from being healthy to becoming ill ($p_{12}$) is typically higher than the probability of transitioning from illness to death ($p_{23}$). Moreover, the likelihood of an immediate progression from being healthy to death ($p_{13}$) without going through the illness state is usually very small. This is expressed mathematically as $p_{12}> p_{23} > p_{13}$ , and these probabilities sum to 1, as follows:

19 $$\begin{aligned} p_{12} + p_{23} + p_{13} = 1. \end{aligned}$$

In a multistate survival model , these transition probabilities can represent real-world clinical scenarios. For instance, in a longitudinal study of cardiac disease patients, the likelihood of transitioning from a steady state (e.g., state 1: asymptomatic) to a more severe state (e.g., state 2: symptomatic) might be much higher than transitioning directly to death (state 3). The survival probability for each individual during these transitions is derived from these transition probabilities. The Kaplan-Meier survival probability for an individual in a particular transition is denoted by $w_{ij}$, where $i \ne j \in \{1, 2, 3\}$, and reflects the likelihood of surviving beyond time $t$ for a given transition. These individual survival probabilities are weighted by group-level factors such as $p_{ij}$, which capture the transition likelihoods within a population cohort (e.g., patients undergoing similar treatments). The modified weights are:

20 $$\begin{aligned} w_{ij}^{'} = w_{ij} \cdot p_{ij} \end{aligned}$$

For instance, consider a cancer study where individuals are grouped by stages of progression. If patients in one group are more likely to progress from stage 1 (initial diagnosis) to stage 2 (advanced disease) before transitioning to stage 3 (death), the transition probabilities between these states can vary based on the group. A patient receiving effective treatment may have a higher $p_{12}$ (transition to advanced disease) but a lower $p_{23}$ (transition to death), while another patient with more aggressive disease might have higher $p_{23}$ values. For each individual, the total weight is computed as:

$$w^{T} = {\left\{ \begin{array}{ll} \sum _{i \ne j} w_{ij}^{'} & \text {if the patient has experienced transitions}, \\ 1 & \text {if the patient has not experienced transitions}. \end{array}\right. }$$

For example, if a heart disease patient transitions from an asymptomatic state to experiencing symptoms (state 1 to state 2) but does not progress to death, their total weight $w^{T}$ would be influenced more by $p_{12}$. If another patient does transition to death, $p_{23}$ would have a larger impact on the total weight, reflecting that different patients experience different transition trajectories based on their condition and treatment outcomes.

Finally, the weighted survival time for an individual is modified as:

21 $$\begin{aligned} \hat{S_{k}}(t)&= \sum _{i \ne j} w_{ij}' S_{ij}(t) \nonumber \\&= w^{T} S_{ij}(t). \end{aligned}$$

This method allows for more precise modeling of survival times by incorporating both sojourn time in each state and the variability in transition probabilities. In clinical studies, such as those investigating chronic diseases like diabetes or cancer, this approach provides a more nuanced prediction of survival outcomes. It accounts for individual differences (e.g., patient age, treatment type) and group-level factors (e.g., the likelihood of progression based on cohort characteristics), offering a more realistic representation of disease progression and patient survival. For instance, in a study of diabetic patients, those with good blood sugar control might have lower transition probabilities from the stable (state 1) to the more severe disease (state 2), compared to patients with poor control, leading to longer survival times in the stable state. By adjusting for these individual and group-level factors, the model delivers better predictions and reflects the complexity of real-world transitions. In this context, a custom code has been developed to generate an MSM with weighted overall survival time.

### Estimation

Frailty models are essential for accounting for unobserved heterogeneity in survival analysis. These models introduce a random effect that modifies the hazard function, reflecting unmeasured risk factors that vary across individuals or groups. In MSMs, frailty is often shared across clusters, representing unobserved factors that influence transitions between states. In this section, we derive the joint survival function for clustered individuals, apply the Laplace transformation to account for frailty, and explain the estimation process using a gamma frailty distribution. The gamma distribution was chosen to model the frailty term due to its mathematical simplicity, interpretability, and widespread application in survival analysis. It allows for a closed-form expression of the marginal likelihood in the Cox proportional hazards framework, facilitating efficient estimation. Although other distributions such as the inverse Gaussian are also used, the gamma distribution offers a flexible and robust approach to capturing unobserved heterogeneity across clusters without significantly affecting inference or model fit^17^.

The proportional hazards equation for the transition from state $i$ to state $j$ $(i \ne j)$ in the presence of frailty is given by:

22 $$\begin{aligned} h_{ij}(t|\textbf{X} = \textbf{x}, Z_{ij}) = Z_{ij}h_{0ij}(t)\exp (\beta ^T \textbf{x}) \end{aligned}$$

Here, $Z_{ij}$ represents the frailty term, which captures unobserved heterogeneity in the transition between states $i$ and $j$. The baseline hazard $h_{0ij}(t)$ is modified by the covariates $\textbf{x}$ and the frailty term. We treat transitions from state $i$ to state $j$ in a MSM as clusters, as is commonly done in shared frailty models. Let $k = 1, 2, \dots , n$ represent the number of individuals in a particular cluster, with the survival time of the $k$-th individual in the $ij$-th transition denoted by $S(t_{ijk})$. The joint multivariate survival function for individuals in the $ij$-th transition, conditioned on the transition-specific frailty $Z_{ij}$ common to all individuals in the cluster, is given by:

23 $$\begin{aligned} S(t_{ij1}, t_{ij2}, \dots , t_{ijn} \mid X_{ij}, Z_{ij})&= S(t_{ij1} \mid X_{ij1}, Z_{ij1}) \cdots S(t_{ijn} \mid X_{ijn}, Z_{ijn}) \nonumber \\&= \exp \left( -Z_{ij} \sum _{k=1}^{n} H_{0}(t_{ijk}) \exp (\beta ^T X_{ijk}) \right) \end{aligned}$$

where $H_{0}(t) = \int _{0}^{t} h_{0}(s) ds$ is the cumulative baseline hazard function, and $X_{ij} = (X_{ij1}, X_{ij2}, \dots , X_{ijn})$ is the covariate matrix of individuals in the $ij$-th transition. In this setup, the frailty term $Z_{ij}$ introduces shared unobserved heterogeneity for individuals within the same transition cluster.

To obtain the marginal survival function, we take the expectation of the joint survival function concerning the frailty variable $Z_{ij}$. This is commonly done using the Laplace transformation, which simplifies the integration over the frailty distribution:

24 $$\begin{aligned} S(t_{ij1}, t_{ij2}, \dots , t_{ijn} \mid X_{ij})&= E\left[ S(t_{ij1}, t_{ij2}, \dots , t_{ijn} \mid X_{ij}, Z_{ij})\right] \nonumber \\&= E\left[ \exp \left( -Z_{ij} \sum _{k=1}^{n} H_{0}(t_{ijk}) \exp (\beta ^T X_{ijk})\right) \right] \nonumber \\&= L\left( \sum _{k=1}^{n} H_{0}(t_{ijk}) \exp (\beta ^T X_{ijk}) \right) \end{aligned}$$

where $X_{ij} = (X_{ij1},X_{ij2},\cdots , X_{ijn})$ is the covariate matrix for the $ij-th$ transition. Here, $L$ represents the Laplace transformation of the frailty variable $Z_{ij}$. The Laplace transformation provides a convenient way to handle the expectation of the survival function concerning the frailty distribution, yielding the marginal survival function. The joint survival function for all time-to-event data across clusters is calculated by multiplying the survival functions of each cluster, assuming independence between clusters.

In frailty models, the frailty variable is often presumed to follow a gamma distribution due to its flexibility and closed-form solutions for the Laplace transformation. We consider a one-parameter gamma distribution with expectation 1 and variance $\sigma ^2$, where the density function of the frailty is:

$$\begin{aligned} f(z_{ij}; \sigma ^2) = \frac{z_{ij}^{1/\sigma ^2 - 1} e^{-z_{ij}/\sigma ^2}}{\sigma ^{2/\sigma ^2} \Gamma (1/\sigma ^2)} \end{aligned}$$

The corresponding Laplace transformation for the gamma distribution is $L(s) = (1 + \sigma ^2 s)^{-1/\sigma ^2}$. Using this, the marginal survival function in equation (27) becomes:

25 $$\begin{aligned} S(t_{ij1}, t_{ij2}, \dots , t_{ijn} \mid X_{ij})&= L\left( \sum _{k=1}^{n} H_{0}(t_{ijk}) \exp (\beta ^T X_{ijk})\right) \nonumber \\&= \left( 1 + \sigma ^2 \sum _{k=1}^{n} H_{0}(t_{ijk}) \exp (\beta ^T X_{ijk}) \right) ^{-\frac{1}{\sigma ^2}} \nonumber \\&= \left( \sum _{k=1}^{n} S(t_{ijk} \mid X_{ijk})^{-\sigma ^2} - (k-1) \right) ^{-\frac{1}{\sigma ^2}} \end{aligned}$$

The conditional likelihood function for clusters of size $n$ is given by:

26 $$\begin{aligned} L(\beta , \sigma ^2) = \prod _{ij} \int _{0}^{\infty } \prod _{k=1}^{n} \left( Z_{ij} h_{0}(t_{ijk}) \exp (\beta ^T X_{ijk})\right) ^{\delta _{ijk}} e^{-Z_{ij} H_{0}(t_{ijk}) \exp (\beta ^T X_{ijk})} f(Z_{ij}; \sigma ^2) dZ_{ij} \end{aligned}$$

where $f(Z_{ij}; \sigma ^2)$ represents the density function of the gamma distribution. The closed form of this integral can be derived by introducing the following simplification:

$$\begin{aligned} y_{ij} = \frac{1}{\sigma ^2} + \sum _{k=1}^{n} H_{0}(t_{ijk}) \exp (\beta ^T X_{ijk}) \end{aligned}$$

Using this substitution, the conditional likelihood from equation (26) simplifies as follows:

27 $$\begin{aligned} L(\beta , \sigma ^2)&= \prod _{ij, i \ne j} \frac{\prod _{k=1}^{n} \left( h_{0}(t_{ijk}) \exp (\beta ^T X_{ijk})\right) ^{\delta _{ijk}}}{ y_{ij}^{\frac{1}{\sigma ^2} + d_{ijk}} \sigma ^{2/\sigma ^2} \Gamma (1/\sigma ^2)} \times \nonumber \\&\quad \int _{0}^{\infty } (y_{ij} z_{ij})^{\frac{1}{\sigma ^2} + d_{ij} - 1} e^{-y_{ij} z_{ij}} d(z_{ij}) \end{aligned}$$

28 $$\begin{aligned}&= \prod _{ij, i \ne j} \frac{\Gamma (1/\sigma ^2 + d_{ij}) \prod _{k=1}^{n} \left( h_{0}(t_{ijk}) \exp (\beta ^T X_{ijk})\right) ^{\delta _{ijk}}}{\left( 1/\sigma ^2 + \sum _{k=1}^{n} H_{0}(t_{ijk}) \exp (\beta ^T X_{ijk}) \right) ^{1/\sigma ^2 + d_{ij}} \sigma ^{2/\sigma ^2} \Gamma (1/\sigma ^2)} \end{aligned}$$

where $\delta _{ijk}$ denotes the individual event status in the $ij-th$ cluster, $d_{ij} = \sum _{ij}\delta _{ijk}$ is the number of events observed in that particular cluster, $z_{ij}$ is the frailty term and $H_{0}(t_{ijk})$ is the cumulative hazard function of the corresponding cluster, $\sigma ^2$ is the frailty variance.

Finally, the unconditional log-likelihood function for the gamma frailty model^6,18^ is expressed as:

29 $$\begin{aligned} \begin{aligned} \log L(\beta , \sigma ^2) =&\sum _{ij} \left[ d_{ij} \log \sigma ^2 + \log \Gamma \left( \frac{1}{\sigma ^2} + d_{ij}\right) - \log \Gamma \left( \frac{1}{\sigma ^2}\right) \right. \\&\left. - \left( \frac{1}{\sigma ^2} + d_{ij}\right) \log \left( 1 + \sigma ^2 \sum _{k=1}^{n} H_{0}(t_{ijk}) \exp (\beta ^T X_{ijk}) \right) \right. \\&\left. + \sum _{k=1}^{n} \delta _{ijk} \left( \beta ^T X_{ijk} + \log h_{0}(t_{ijk})\right) \right] \end{aligned} \end{aligned}$$

In a semi-parametric shared gamma frailty model including covariates, the latent frailty $Z_{ij}$ for each cluster can be estimated as^19^:

30 $$\begin{aligned} \hat{Z_{ij}} = \frac{1/\hat{\sigma ^2} + \sum _{k=1}^{n} \delta _{ijk}}{1/\hat{\sigma ^2} + \sum _{k=1}^{n} H_{0}(t_{ijk}) \exp (\beta ^T X_{ijk})} \end{aligned}$$

In the Cox proportional hazards model with shared frailty, the baseline hazard function is estimated non parametrically using the Breslow estimator. This method accumulates hazard contributions over observed event times and accounts for the number of individuals at risk at each time point. Since the Cox model does not assume a specific form for the baseline hazard, it remains unspecified and is derived directly from the data. In the case of multiple transitions, separate Cox frailty models are fitted for each transition, and the baseline hazard is estimated independently for each one using this approach.

## Simulation

### Data generation

Generating simulated data that closely mimics real-world events is essential to evaluate the effectiveness of novel or established statistical techniques for survival analysis. These simulated datasets are utilised to evaluate the influence of several prognostic factors and transition probabilities in MSMs, as well as to quantify the uncertainty of model predictions, including frailty effects across different transitions within the MSM. Here we considered two different states in a multistate model and generated individual survival times, $stime_{13}$ and $stime_{23}$, and corresponding statuses, $status_{13}$ and $status_{23}$.

Suppose *T* represents the event time for the transition from *i* to *j*. We assumed the baseline hazard $h_{ij0}(t)=\lambda \gamma t^{\gamma -1}$ is obtained from event times following a Weibull distribution with scale and shape parameters $\lambda ,\gamma$ respectively. The hazard at event time *t* is given by,

31 $$\begin{aligned} h_{ij}(t|\textbf{X})=h_{ij0}(t) e^{\textbf{x}\varvec{\beta }} \end{aligned}$$

For the Weibull distribution baseline hazard is given by $h_{0}(t) = \lambda \gamma t^{\gamma -1}$, and the survival function is $S(t|\textbf{X})=e^{-H_{ij}(t|\textbf{X})}$ where $H_{ij}(t|\textbf{X}) = \lambda t^{\gamma }e^{\textbf{x}\varvec{\beta }}$ is the cumulative hazard.

Since the survival probability lies between 0 and 1, we assume $S(t) \sim U(0,1)$. Then we generated the survival time distribution using a uniform random variable defined by a time function,

32 $$\begin{aligned} t = H_{0}^{-1}[-log(u) exp(-\beta \textbf{x})] \end{aligned}$$

Now putting $H_{ij0}(t)=\int _0^t h_{0ij}(t)dt=\lambda t^{\gamma }$ in equation(32), we obtain the simulated survival time with frailty effect for the transition from *i* to *j* as

33 $$\begin{aligned} t= \left[ \frac{-log u}{\lambda e^{\textbf{x}\varvec{\beta }}} \right] ^{1/\gamma } \end{aligned}$$

**Fig. 2:**
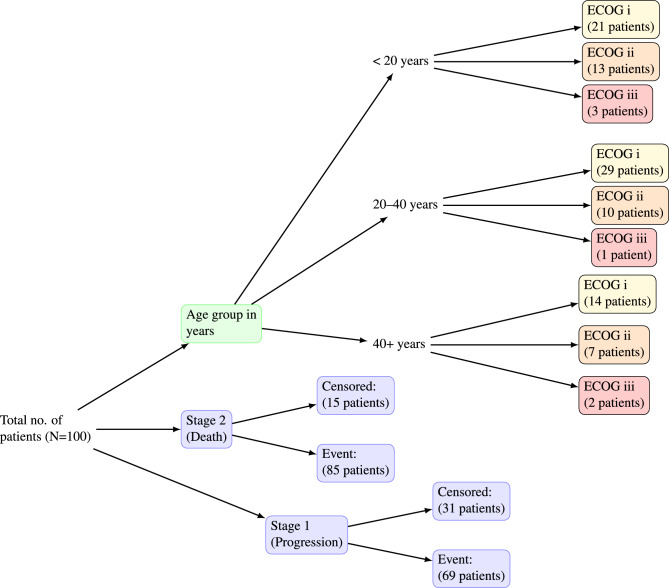
Data description of the example MSM dataset with progression and death states.

Here the random variable *u* represents uniform distribution which is used to simulate survival time. The covariates that we have considered here say $X_{i}$s are both continuous and dichotomous at the baseline level and randomly generated following a normal and binomial distribution and independent of each other. In numerous cancer research, the predictive significance of post-operative carcinoembryonic antigen (CEA) level in individuals with advanced and metastatic malignancies has been utilized as a significant covariate^20,21^. Studies indicate that continuous assessment of gene expression or mutation load (e.g., proportion of cells exhibiting EGFR or KRAS mutations) is employed in molecular investigations of lung cancer to predict responses to targeted treatments^22,23^. Hence, we have considered $X_{1}$ and $X_{2}$ to be baseline CEA level and baseline EGFR level. Both the variables are generated randomly following normal distribution such as: $X_{1} \sim N(0,1)$ and $X_{2} \sim N(10,20)$. The ECOG status was the Eastern Cooperative Oncology Group performance status, a metric employed to evaluate a cancer patient’s functional capacity and general health^24,25^. The ECOG status is typically assessed on a scale from 0 to 5 to evaluate eligibility for clinical trials and to forecast prognosis. In our study, we have assumed the random variable $X_{3}$ as categorical, representing the ECOG status of patients. However, we have examined just three levels of this variable: (*I*, *II*, *III*), with proportions of 60%, 30%, and 10%, respectively. The random variable $X_{4}$ is categorical, corresponding to age groups with three distinct categories: under 20, 20-40, and 40 and above, with proportions of 30%, 50%, and 20%, respectively. The data description of the simulated data is given in Fig. 2 and the summary statistics is presented in Table 1.

In this regard R function **simmsm** has been developed to simulate multistate survival data using cox PH model and a parametric baseline hazard. We applied the above procedure to simulate the survival time for the transition from state(1) to state(3), state(2) to state(3) denoted by $\zeta _{13}$ and $\zeta _{23}$ respectively. Since the frailty term *Z* follows a Gamma distribution with unit mean, we considered the scale and shape parameters to be the same. The parameter values for the given equation for survival time $stime_{13}$ were chosen arbitrarily as follows $\beta _1= -0.05, \beta _2=0.01, \beta _3=0.02, \beta _4=0.01, \gamma =1.5, \lambda =0.1$. Similarly, for survival time $stime_{23}$ the parameter values were chosen to be $\beta _1= -0.03, \beta _2=0.02, \beta _3=-0.02,\beta _4=0.01, \gamma =2,\lambda =0.2$. The survival time equation (33) was used to generate the entire dataset, with two different survival times, $stime_{13}$ and $stime_{23}$, and the corresponding statuses. The censoring time follows an exponential distribution with a rate parameter of 0.1. Comparing the survival time and the censoring time the status for each state is generated. Then the statuses of the two transitions were compared, and the corresponding survival times $stime_{13}$ and $stime_{23}$ were adjusted, resulting in the generation of two sets of transition times for transitions $\zeta _{13}$ and $\zeta _{23}$ respectively. A total of 100 such datasets were simulated containing 500 individuals each.

### Analysis

Our approach involves estimating frailty effects in multistate models for specific transitions, which accounts for the variability among individuals in the specific cohort. The data used in this study includes three states in the multistate model. Using Cox Ph model coefficients of the covariates and frailty variances were generated for each transition for the simulated dataset given in table 2. Frailty at group level in survival analysis pertains to unobserved random factors or latent variables that explain the variability in the likelihood of an event (such as death) among people in a study. In order to account for the variance at the subject level in the dataset, we generated a new survival time by multiplying the individual-specific weights with the original survival time of each individual.

For the simulated dataset with original OS, the frailty variance was highest in transition $\zeta _{23}$ (2.83), indicating substantial latent heterogeneity among study groups for that transition. In contrast, $\zeta _{12}$ had the lowest frailty variance (0.25), suggesting relatively homogeneous risk across clusters. After applying weighted survival times, frailty variance decreased in $\zeta _{13}$ (now 0.29), while $\zeta _{23}$ remained the most heterogeneous (10.05). This suggests the weighting procedure successfully reduced unobserved variation in $\zeta _{13}$ but not in $\zeta _{23}$.

At the group level, these variances quantify how much baseline hazard rates vary across latent clusters, likely research centers, demographic strata, or unmeasured categories beyond what observed covariates explain. A large frailty variance (e.g., 2.83 or 10.05) implies strong clustering effects: some groups face far higher or lower risk than the average. Such clustering can induce dependence among individuals within the same group, often quantified by Kendall’s $\tau$; for gamma frailty, the relationship is $\tau = \theta / \theta +2$, so $\theta =2.83$ translates to $\tau \approx 0.59$, meaning moderate to strong within-group correlation. Furthermore, when the frailty variance is high, the groups with higher risk tend to have events earlier, leaving behind lower-risk groups. This changes the overall risk pattern over time. The reduction of frailty variance for $\zeta _{13}$ suggests the weighting procedure effectively homogenized hazards across clusters for that transition. Importantly, reduced frailty variance after weighting indicates that bias in transition specific covariate effects has been addressed, particularly in $\zeta _{23}$, where covariate biases decreased. This aligns with the theory showing that unobserved heterogeneity attenuates regression coefficients if left unmodeled or unweighted. A boxplot illustrating the estimated bias for each covariate across different transitions comparing unweighted and weighted simulation methods has been included in Fig. 5. Similar visualizations can also be generated for MSE and other estimation metrics.

## Analysis of EBMT platelet recovery data

The proposed method was also applied to the European Society for Blood and Marrow Transplantation (EBMT) dataset, referred to as the ebmt3 dataset, accessible within the “mstate” package. This dataset contains information on 2,204 people who received transplant treatment from 1985 to 1998. The dataset has a unique patient identifier, two distinct states, a status, and four covariates. The data includes information on the survival of patients with blood cancer after transplant treatment. The two intermediate states are the duration until platelet recovery and the duration until recurrence or mortality. Transplant is the initial state, and the time from transplant until platelet recovery or death is calculated. A total of 2204 patients who were treated between 1985 and 1998 were included in the study. Four prognostic factors, namely disease subclassification (dissub), age, donor-recipient gender match (drmatch), and T-cell depletion (Tcd), were measured at the baseline level for all patients. All these covariates were treated as time-fixed categorical covariates. A new categorical covariate representing the treatment “arm” was randomly created from a binomial distribution and added to the existing dataset for analysis and for comparison purposes.

The Cox Ph model was applied to each transition in the dataset separately. The results of the proportional hazard model on the ebmt3 dataset along with transition-specific frailty variances, are presented in Table 3. The frailty variance was highest for the transition $\zeta _{13}$ followed by $\zeta _{23}$ and $\zeta _{12}$ in the dataset with original OS 3. A higher frailty variance in transition $\zeta _{13}$ indicates greater unobserved heterogeneity, which means individuals differ more strongly in their risk of making that specific transition due to latent factors, and the frailty variance has been reduced for weighted overall survival. The weighted EBMT dataset, created by multiplying the original survival times by individual-specific weights, exhibited the same trend in frailty variance. Frailty variance is lowest for $\zeta _{12}$ in the weighted EBMT dataset which means the risk is relatively homogeneous across individuals. The measured covariates likely capture most of the relevant variation in risk for that transition. The standard error (SE) of the coefficients has also been reduced in transition $\zeta _{12}$ and $\zeta _{13}$.

**Fig. 3:**
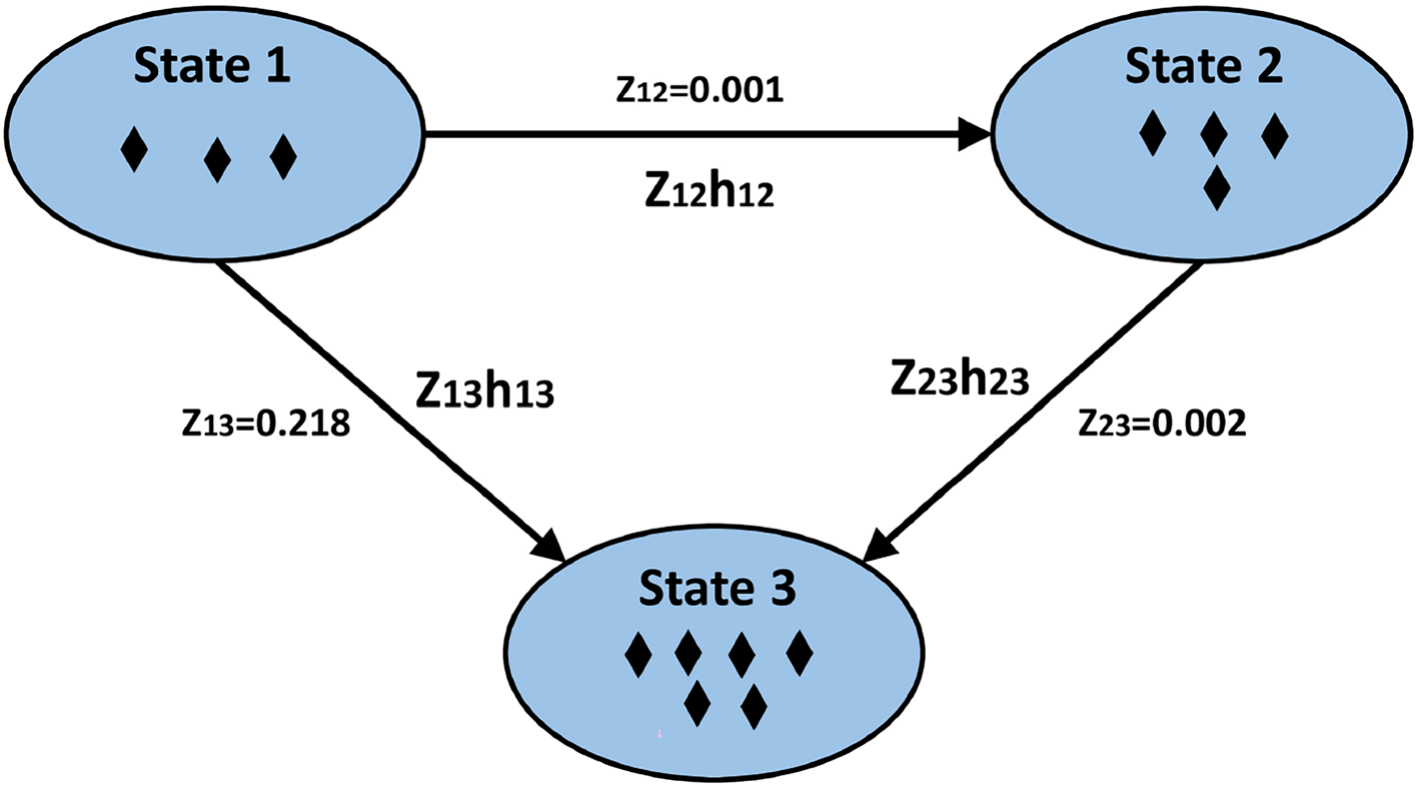
Multi state model with three states and transition-specific frailty $Z_{12}, Z_{23}, Z_{13}$ along with frailty estimates.

**Fig. 4:**
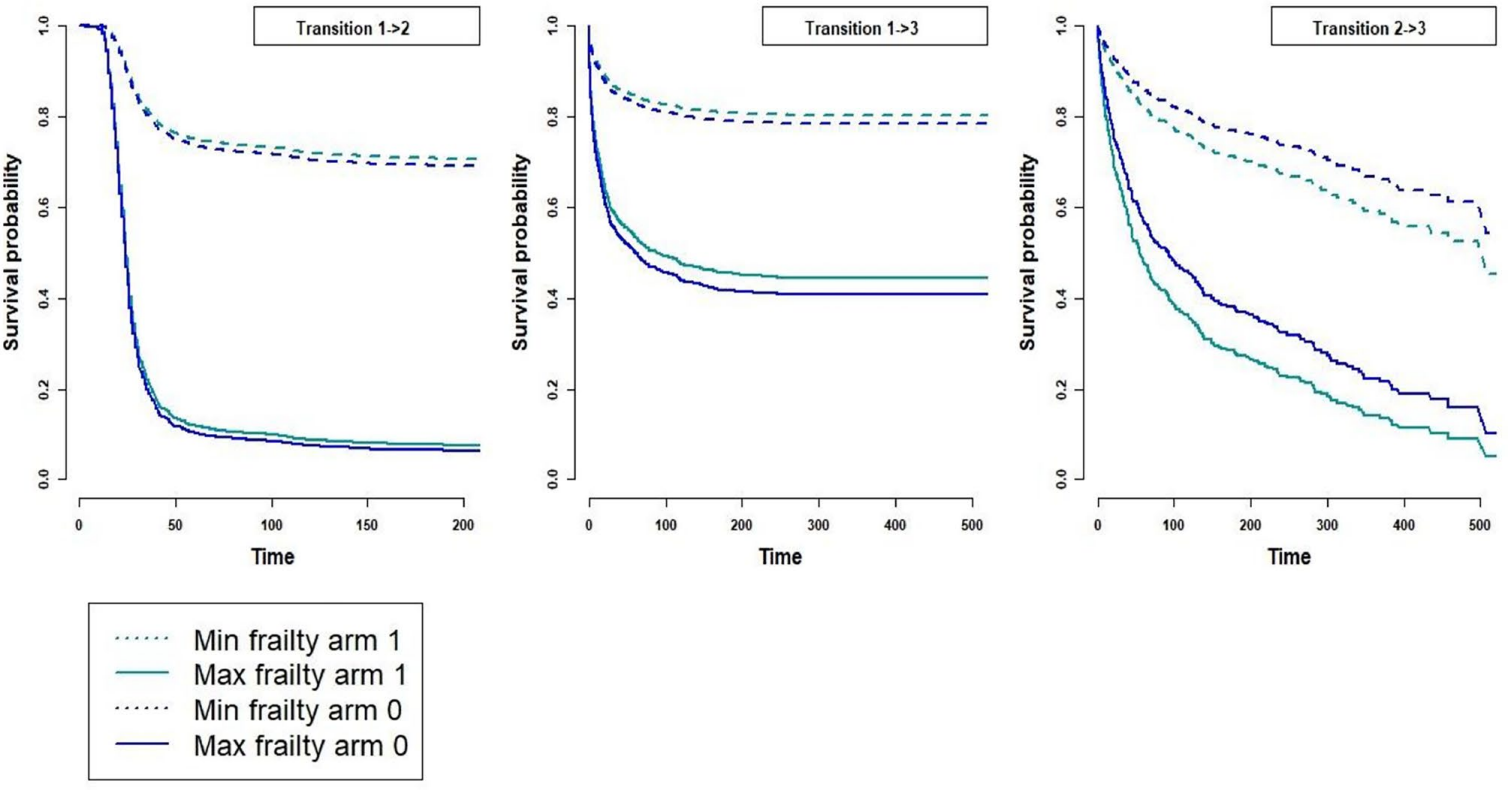
Survival probability curves for transitions $\zeta _{12}$, $\zeta _{13}$, and $\zeta _{23}$ in treatment arms 0 and 1, comparing maximum (1.3) and minimum (-0.7) frailty values.

The survival probability curve for each transition in both treatment arms was based on anticipated maximum and lowest frailty values, has been displayed to illustrate the variations in individual survival across different arms for different frailty values. The plotted curve is given in Fig. 4. For example, in the transition 1$->$2, the survival curve has been fitted for two different frailty values, 1.3 and -0.7, for both arm 0 and arm 1. The solid line represents the survival curve for a frailty of 1.3, and the dashed line represents a frailty of -0.7. Similarly, for transition 1$->$3 and 2$->$3 the maximum and minimum frailty value for an individual is taken to be 1.2 and -0.109 respectively for illustration purposes. It can be seen that survival has decreased with higher frailty. However, for the transition 1$->$3 and 2$->$3, a difference in survival between the two arms can also be seen for different frailty values.

## Discussion

The incorporation of frailty effects into multistate modeling represents a pivotal advancement in survival analysis, particularly when investigating the dynamics of health transitions across various populations. Frailty refers to unobserved heterogeneity among individuals that influences their risk of transitioning between different health states, such as recovery, deterioration, or death^26,27^. By integrating frailty into MSMs, researchers can enhance predictive accuracy, accounting for individual differences that standard models may overlook. For instance, a significant study focusing on nosocomial pneumonia in intensive care units highlighted how frailty models outperform traditional MSMs, as depicted in Fig. 3. The addition of frailty components led to improved predictive performance, particularly in clinical settings where patient outcomes are influenced by unobserved factors^28^. Similarly, Gao et al. demonstrated the impact of frailty on life expectancy estimates within a Chinese cohort. Their multistate model revealed that frailty-free life expectancy is considerably higher among individuals without frailty, underscoring the importance of dynamically modeling frailty to reflect changes in health status over time^29^. Moreover, Abdullah et al. introduced a parametric frailty model that incorporates time-dependent covariates, emphasising modelling its superiority over models without frailty effects in providing more accurate survival estimates. This finding is particularly relevant for clinical trials and longitudinal studies, where capturing the effects of various covariates on survival is critical^12,30–32^. However, Putter et al. and Rotolo et al. critically assessed the necessity of frailty in MSMs, arguing that while frailty can complicate interpretation, it remains essential for capturing underlying heterogeneity in survival data. Their exploration into identifying frailty effects supports the argument for its inclusion in survival models^3,33^. Frailty models are commonly applied as time-dependent random effect models, where the random effect exerts a multiplicative influence on the hazard function. In univariate survival analysis, mixture likelihood models are often employed to address frailty impacts, particularly when sufficient variation in regressor variables exists^34,35^. However, the estimation of frailty distributions is not always possible, as highlighted by Hougaard^36,37^. The choice of frailty distribution, such as gamma or inverse Gaussian, can substantially influence parameter estimates. Disregarding frailty, particularly when it has a finite mean, may lead to biases toward zero in parameter estimations^38–40^. The incorporation of frailty into multistate models for multivariate survival data allows researchers to model dependencies between events, assuming event times are independent when conditioned on individual random effects. The proposed model in this article, employing a three-stage multistate framework, identifies frailty effects and dependencies across transitions. Future research could extend this model by incorporating more flexible frailty distributions to generalize its application further. In summary, the inclusion of frailty in multistate models significantly enhances the predictive power of survival analyses by accounting for unobserved heterogeneity. Studies consistently demonstrate that frailty models outperform traditional ones, offering deeper insights into health transitions and outcomes. This approach is particularly valuable in clinical settings, where understanding patient health trajectories is crucial for intervention and management.

## Conclusion

This study examines the impact of transition-specific frailties on understanding relationships between stages in a multistate model and identifies transitions with higher heterogeneity among populations. This article uses individual-level weights calculated from their transition probabilities from one state to another state as a factor in determining adjusted survival time. The objective of this study was to analyze the influence of transition-specific frailties in comprehending and quantifying the relationship between various clusters in a MSM with weighted survival time. As a potential future direction, this three-stage MSM can be expanded to create a more extensive model for dynamic predictions and also for competing risk models.

**Table 1:** Summary Statistics of Simulated Dataset.

Survival Time & Covariates	Summary Statistics
Progression Free Survival (time1)	
Median (Min, Max) Time	3.91 (0.21,9.48) years
Overall Survival (time2)	
Median (Min, Max)	5.91 (0.59,16.54) years
Carcinoembryonic Level (CEA, x1)	
Mean (Min, Max)	0.79 (0.003,2.11)
EGFR Expression (x2)	
Mean (Min, Max)	18.19 (0.06,56.93)
ECOG (x3)	
Grade I	64 (60%)
Grade II	30 (30%)
Grade III	6 (10%)
Age Group (x4)	
<20 years	37 (40%)
20-40 years	40 (40%)
40 years & above	23 (20%)

**Table 2:** Simulation results of 100 datasets consist of 500 subjects each for both unweighted and weighted survival time.

Covariates	$\zeta _{12}$		$\zeta _{13}$		$\zeta _{23}$	
	Mean(MSE)	Bias	Mean(MSE)	Bias	Mean(MSE)	Bias
Simulation result of datasets with unweighted overall survival						
CEA level $(\hat{\beta _{1}})$	-0.05(0.03)	-0.01	-0.00(0.01)	0.02	-0.03(0.03)	-0.01
EGFR level$(\hat{\beta _{2}})$	0.01(0.00)	0.01	0.01(0.00)	-0.01	0.02(0.00)	-0.01
ECOG level $(\hat{\beta _{3}})$	0.01(0.05)	-0.01	-0.04(0.01)	-0.02	-0.03(0.07)	-0.01
Age grp $(\hat{\beta _{4}})$	-0.00(0.01)	-0.01	0.01(0.01)	-0.01	0.03(0.17)	0.01
Frailty Est.($\sigma ^2$)	0.25		0.53		2.83	
Simulation result of datasets with weighted overall survival						
	$\zeta _{12}$		$\zeta _{13}$		$\zeta _{23}$	
CEA level $(\hat{\beta _{1}})$	-0.04(0.01)	0.01	-0.01(0.01)	0.02	-0.09(0.15)	-0.06
EGFR level$(\hat{\beta _{2}})$	0.002(0.00)	-0.01	0.01(0.00)	-0.01	0.02(0.00)	-0.01
ECOG level $(\hat{\beta _{3}})$	0.04(0.03)	0.01	-0.03(0.01)	-0.01	-0.17(0.12)	-0.14
Age grp $(\hat{\beta _{4}})$	-0.02(0.05)	-0.03	0.01(0.01)	-0.01	-0.04(0.15)	-0.05
Frailty Est.($\sigma ^2$)	0.43		0.29		10.05	

NB: $\hat{\beta _{1}}$ = estimated baseline CEA level, $\hat{\beta _{2}}$ = estimated baseline EGF gene expression level, $\hat{\beta _{3}}$= estimated coefficient of ECOG status, $\hat{\beta _{4}}$= estimated coefficient of age group

**Table 3:** Results of proportional hazard model for different transitions for unweighted and weighted survival time of ebmt dataset.

Covariate	$\zeta _{12}$		$\zeta _{13}$		$\zeta _{23}$	
	Coef($SE^2$)	HR(CI)	Coef($SE^2$)	HR(CI)	Coef($SE^2$)	HR(CI)
Analysis result of ebmt dataset with unweighted overall survival						
dissubALL	-0.05(0.08)	0.96( 0.82,1.11)	0.27(0.14)	1.31(0.10,1.73)	0.13(0.15)	1.14(0.85,1.52)
dissubCML	-0.30(0.07)	0.74(0.65,0.85)	-0.01(0.11)	0.99(0.80,1.24)	0.26(0.12)	1.29(1.03, 1.63)
Age 20-40	-0.16(0.08)	0.85(0.73,0.99)	0.27(0.16)	1.31(0.97,1.78)	0.07(0.15)	1.07(0.79,1.44)
Age >40	-0.09(0.09)	0.91(0.77,1.08)	0.55(0.16)	1.74(1.26,2.39)	0.58(0.16)	1.79(1.31, 2.45)
drmatch	0.04(0.07)	1.05(0.92,1.19)	-0.10(0.11)	0.91(0.72,1.14)	0.17(0.11)	1.19(0.95, 1.49)
Tcd	0.42(0.08)	1.54(1.31,1.80)	0.31(0.16)	1.37(1.01,1.87)	0.20(0.13)	1.22(0.95,1.57)
Arm	0.01(0.06)	1.00(0.89,1.13)	0.15(0.10)	1.16(0.95,1.41)	-0.07(0.10)	0.93(0.76, 1.14)
Frailty Estimate ($\sigma ^2$)	0.001		0.218		0.002	
Analysis result of ebmt dataset with weighted overall survival						
dissubALL	-0.05(0.08)	0.95(0.81,1.12)	0.27(0.12)	1.31(1.04,1.64)	-0.19(0.20)	0.83(0.56,1.24)
dissubCML	-0.29(0.07)	0.75(0.65,0.86)	0.07(0.09)	1.07(0.89,1.29)	0.44(0.15)	1.55(1.16,2.07)
Age 20-40	-0.13(0.08)	0.88(0.75,1.03)	0.37(0.13)	1.44(1.11,1.86)	-0.03(0.19)	0.97(0.67,1.40)
Age >40	-0.07(0.09)	0.93(0.77,1.11)	0.70(0.14)	2.02(1.54,2.64)	0.43(0.20)	1.54(1.05,2.28)
drmatch	0.01(0.07)	1.00(0.87,1.15)	-0.04(0.09)	0.96(0.79,1.16)	0.17(0.14)	1.18(0.89,1.57)
Tcd	0.48(0.08)	1.62(1.37, 1.91)	-0.17(0.13)	0.85(0.65, 1.10)	0.44(0.15)	1.55(1.15, 2.07)
Arm	0.04(0.06)	1.04(0.92,1.18)	0.08(0.08)	1.09(0.92,1.29)	-0.09(0.13)	0.91(0.70,1.18)
Frailty Estimate ($\sigma ^2$)	0.001		0.124		0.001	

NB: $\zeta _{12}$= Transition from state 1$->$2, $\zeta _{13}$= Transition from state 1$->$3, $\zeta _{23}$= Transition from state 2$->$3

**Fig. 5:**
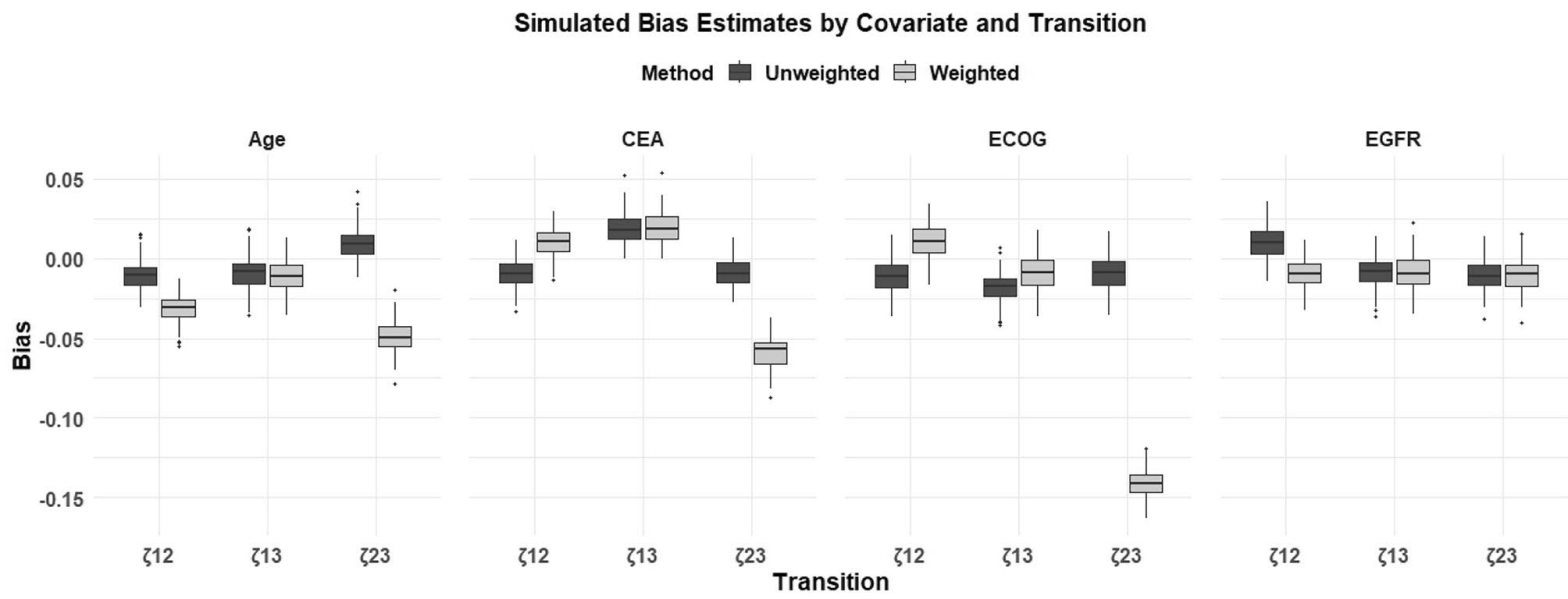
Boxplot of bias in estimated coefficients for each covariate across transitions ($\zeta _{12}$, $\zeta _{13}$, $\zeta _{23}$), comparing weighted and unweighted simulation methods. Each box summarizes the distribution of bias values obtained over multiple simulation replicates, highlighting the performance differences between methods across covariates.

## Data Availability

The dataset can be accessed via the following link: GitHub repository.
